# Why flies look to the skies

**DOI:** 10.7554/eLife.68684

**Published:** 2021-04-16

**Authors:** Stanley Heinze

**Affiliations:** Lund Vision Group and NanoLund, Lund UniversityLundSweden

**Keywords:** vision, central complex, navigation, polarized light, two-photon calcium imaging, circuits, *D. melanogaster*

## Abstract

Fruit flies rely on an intricate neural pathway to process polarized light signals in order to inform their internal compass about the position of the Sun.

**Related research article** Hardcastle BJ, Omoto JJ, Kandimalla P, Nguyen BM, Keleş MF, Boyd NK, Hartenstein V, Frye MA. 2021. A visual pathway for skylight polarization processing in *Drosophila*. *eLife* **10**:e63225. doi: 10.7554/eLife.63225

Imagine you are driving across a desert and you decide to stop and take a walk to stretch your legs. You get lost and cannot see your car – indeed all you can see is sand below and blue sky above. Luckily your phone has GPS so you will be able to find your way back to your car. Walking back you notice that the ants running back and forth across the sand seem to know perfectly well where they are going. How can this be?

Scientists have been asking this question for decades. Back in the 1920s, after observing the navigational abilities of desert ants for the first time, the Swiss entomologist Felix Santschi performed a series of elegant experiments which revealed that these ants can use the Sun to help them navigate their way back to their nests (Wehner, 1990). Moreover, when he blocked the Sun and reflected a patch of blue sky for the ants to see, they changed the direction they were walking in. While Santschi was not able to work out which feature of skylight provided the ants with directional information, Karl von Frisch later demonstrated that bees can perceive the polarization pattern of skylight and use it to navigate (von Frisch, 1949). The same was also found to be true for ants (Wehner, 1997).

Polarization is a feature of light, just as color and intensity are features of light, but it is invisible to the human eye. For a plane wave of light the polarization is defined by the direction of the plane in which the electric field of the wave (its E vector) is oscillating: this polarization can be horizontal, vertical or any angle in between. While direct sunlight contains all possible angles of polarization, sunlight that has been scattered before reaching us – such as light from the sky – is polarized. Moreover, the angle of polarization is directly related to the Sun’s position: this means that a patch of blue sky contains directional information, even if the Sun itself is not visible (Figure 1).

**Figure 1.**
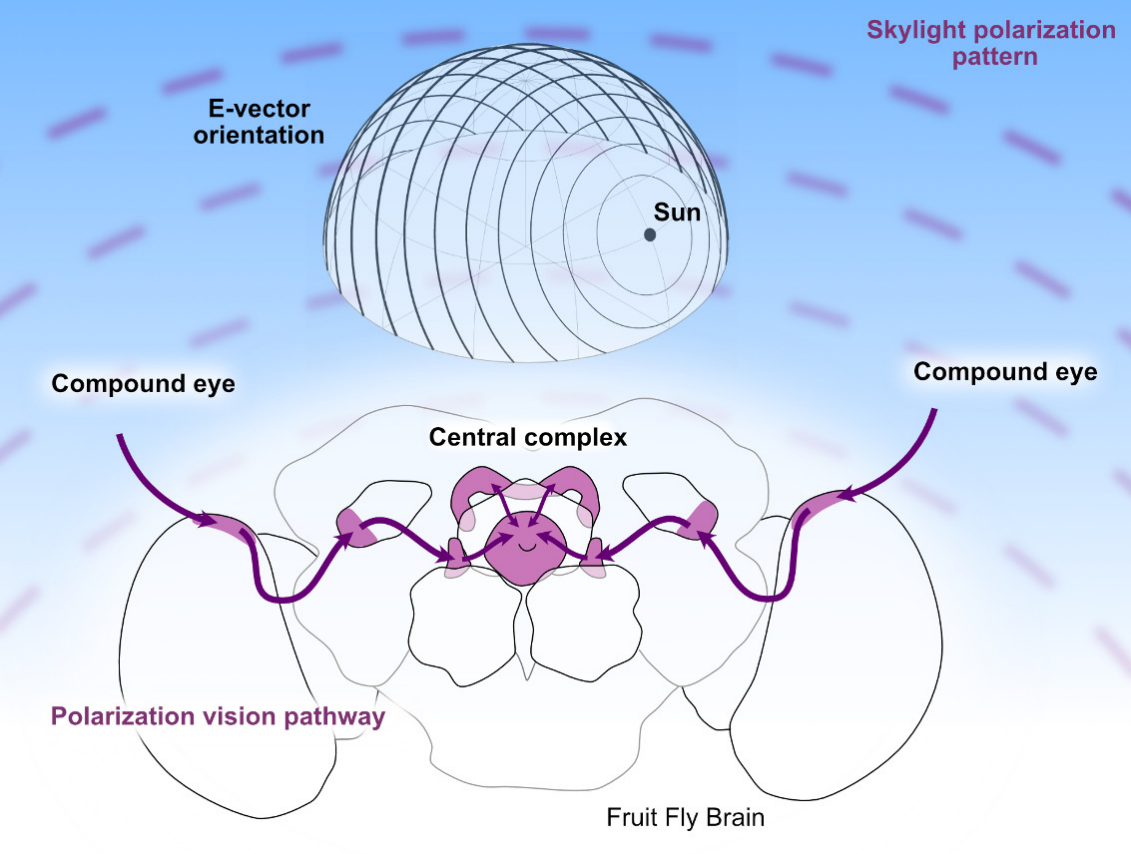
The polarization vision pathway in the brain of *Drosophila.* *Top:* Light from the Sun is polarized when it is scattered by the sky. When the angle of polarization is plotted at different positions on the sky, it forms concentric circles around the Sun (also shown in the background of this figure). Therefore, if an organism can determine the polarization pattern of light from the sky, it can use this information to navigate, even if it cannot see the Sun. *Bottom:* In *Drosophila* information about the polarization of light is collected by specialized structures in the dorsal rim area of the eye (not shown) and then transmitted along a dedicated pathway (purple) to the central complex, where it is combined with a representation of the fly’s heading based on the visual panorama. The signals from each eye pass through the dorsal rim region of the medulla, the anterior optic tubercle, and then through the bulbs before reaching the central complex.

It has been known since the 1980s that insects can detect the E vectors of polarized light with specialized structures in the dorsal rim area of their compound eyes (Labhart and Meyer, 1999). While behavioral responses to polarized light have been widely studied in ants and bees, the neural processing pathways in the brain have been illuminated mostly in locusts. The information about polarization from the dorsal rim area passes through the primary visual processing centers and several other brain regions, before it reaches the central complex in the center of the brain (Homberg et al., 2011). In this region, all this information is combined to encode the current orientation of the locust with respect to the Sun in an array of neurons called head direction cells (Heinze and Homberg, 2007).

Parts of this polarization vision pathway have been confirmed in monarch butterflies, crickets, dung beetles and bees, but not in the fruit fly *Drosophila melanogaster*, even though superb genetic tools are available to study this species. This was puzzling because the head direction neurons in the central complex – also called compass neurons – have been seen more clearly in *Drosophila* than in any other insect species (Seelig and Jayaraman, 2015). However, experiments suggested that the internal compass in *Drosophila* appeared to rely on local visual landmarks rather than a global reference frame like the sky. Now, in eLife, Ben Hardcastle and co-workers at UCLA report the results of experiments that shed new light on the way that information about polarization is processed in the brain of *Drosophila* (Hardcastle et al., 2021).

Most of the species in which polarized-light vision was studied previously (either behaviorally or neurally) were expert navigators. Monarch butterflies, for example, can migrate over thousands of kilometers, while desert ants are able to locate a hidden nest after convoluted foraging excursions in novel territory. Flies do neither – which suggests that they might not need to rely on a global reference frames such as the sky in order to navigate. However, recent work showed very clearly that even the tiny fruit fly has a remarkable ability to maintain a straight course for many kilometers (Leitch et al., 2020), that it can use polarized light to do this (Warren et al., 2018), and that this light is indeed detected by a dorsal rim area in its compound eyes (Weir et al., 2016).

Hardcastle et al. have now completed the rest of the story, essentially recapitulating in *Drosophila* decades of work done on other insects. Using in vivo imaging of brain neurons engineered to express a calcium indicator that lights up when neurons are active, the UCLA team monitored the activity of neurons along the polarization vision pathway when showing light of different polarization angles to the flies. The result is a manuscript filled with stunning details of a neural processing pathway that was meticulously dissected anatomically and functionally, beautifully demonstrating how polarized light information is reshaped at each processing stage. Moreover, the pathway uncovered by the UCLA team looks remarkably similar to its counterparts in other insect species, all the way up to the central complex, where there are surprising differences.

In the central complex, we are left with a fascinating disparity between species, and also between head direction codes based on landmarks and head direction codes based on polarized light. As we understand today, the central complex integrates inputs from all available sources (celestial cues, the visual panorama, even the wind) to unambiguously encode the orientation of the animal in space in an ordered array of head direction cells. Locusts use polarized light to produce a "heading map" that covers 180° of azimuthal space (Heinze and Homberg, 2007), whereas flies rely on visual cues from landmarks to produce an internal compass that covers 360° (Kim et al., 2019).

Hardcastle et al. now report that the representation of polarized light in the central complex in flies appears to follow neither of these models. Indeed, the E vectors are not mapped in any systematic way at all. Rather, neurons across the central complex appear to encode only two E vector angles, separated by 90°. How can the same neurons use the visual panorama to encode body angle with respect to 360° of space, if they simultaneously encode only "forward versus sideways" when processing polarized light? Hence the paper by Hardcastle et al. does what all great papers should do; it leaves us with new questions. Are global and local reference frames indeed integrated into the same internal compass signal? Are these integration principles different in species with distinct navigational demands? Luckily, Hardcastle et al. also provide a very good idea of where to look for the answers to these questions!
